# Passing-skill training vs. small-sided games for improvement of side-foot kick accuracy among youth female soccer players

**DOI:** 10.3389/fspor.2025.1506563

**Published:** 2025-04-17

**Authors:** Tomas Carlsson, Joakim Broman, Jenny Isberg, Magnus Carlsson

**Affiliations:** School of Health and Welfare, Dalarna University, Falun, Sweden

**Keywords:** football, performance, test, skill acquisition, adolescents

## Abstract

This study compared the effectiveness of a passing-skill training (PST) concept (theory lectures, passing-skill training, and external- and internal-focus feedback) and three-a-side small-sided games (SSG) on improving side-foot kick accuracy among youth female soccer players. Seventeen youth female soccer players (age: 12.7 ± 0.8 years) completed the pre-post-intervention study, where the pre- and post-tests included three 10-m passing accuracy tests with increasing level of complexity: (1) stationary ball with a fixed target (SBFT), (2) rolling ball with a fixed target (RBFT), and (3) rolling ball with a moving target (RBMT). Based on the pre-tests, the participants were matched into the PST group or the SSG group. The training consisted of nine 30-min sessions for both groups during a period of 4.5 weeks. During the intervention, the PST group significantly improved the side-foot kick accuracy across all three tests. In contrast, the SSG group showed no significant change in passing accuracy in either the RBFT test or the RBMT test; however, their side-foot kick accuracy was significantly reduced in the SBFT test. There were significant between-group differences in side-foot kick accuracy across all three tests after the intervention, where the PST group had a greater side-foot kick accuracy than the SSG group. In conclusion, results indicate that the PST concept significantly enhances passing accuracy across all tested variables, unlike SSG, suggesting PST's superiority in technical skill development.

## Introduction

**S**hort on-the-ground passes has previously been found to be the most frequently used passing activity in elite soccer (1–3). Execution of accurate side-foot kicks is essential for retaining possession of the ball by the team (4), and analyses of games of the group stages of FIFA World Cup 2014 showed that match statistics variables related to passing, such as ball possession, number of short passes, and average pass streak, had a positive effect on the probability of winning (5). In line with these findings, the ability to execute an accurate pass has been accentuated in elite soccer, where the percentage of successful passes increased between seasons 2006–07 and 2012–13 in the English Premier League (6), and the percentage of successful passes was significantly higher in Premier League than the leagues closest under (i.e., Championship and League 1) (7). Another approach to establish the passing effectiveness is to investigate the variables “space control” (i.e., teammate's distance to the closest defender when he/she receives the pass) and “number of outplayed players” (i.e., decrease of the number of defenders between the teammate that receives the pass and the goal) during matches; it was found that teams scoring more goals on average also outplayed more players and were more successful in increasing space control in the attacking third through passing (8). To achieve these advantages of greater space control and number of outplayed players by effective passing, it is necessary to have a great passing accuracy within the team. The importance of having a high passing effectiveness within a team was emphasised in a previous study, where the team was approximately 24% more likely to win games if their mean percentage of successful passes was improved by 1% (9). Therefore, it is of paramount importance to improve the soccer players' side-foot kick accuracy to improve the performance capacity of the team.

The ability to execute accurate side-foot kick passes has been found to differentiate both male (10) and female (11) soccer players with regard to competitive standard. Competitive-standard differences in passing accuracy has also been found within different age groups of youth male soccer players (12). Recently, it was reported that passing skills in soccer develop with age between the age of 10 and 14 years; however, the passing skills differed between genders in all age groups, where boys had a superior skill compared to girls (13). Advanced biological maturity status has been shown to be associated with slightly better performance in some technical-skill tests (e.g., ball control with the body and head, and dribbling with a pass), but maturity was not a predictor of passing accuracy (14). Previously, it was reported that U-13 players slightly later in maturation but with better soccer skills had a higher prevalence of being an active soccer player 10 years later than their more matured and less skilled counterparts (15). Moreover, they also showed that the U-15 players' soccer skills (i.e., ball control and passing accuracy) at baseline were indicators of later playing status (regional vs. national level) (15). The importance of being technical skilled as a youth soccer player is also emphasize by the results in a study where the variables net hope, motor abilities, technical skills and biological maturity were analysed once a year for three years from the age of twelve; the researchers found that players with a technical skill classified as “high”, and scoring above average for the other three variables, were more likely to reach the highest level of performance (16). Technical skill in youth soccer players has also been highlighted as one potential predictor of adult high performance (17). Based on the results presented above, it appears that soccer players should acquire fundamental technical skills at a relatively high level at an early stage. A key basic skill for youth soccer players to master independent of level of expertise (elite or novice) is short passes (≤11 m) (18), and to develop this technical skill it is necessary to focus on passing-skill training.

An advantage in this sense is the great trainability of young soccer players' motor performance and technical skills. A study including 600 elite young soccer players found that the greatest improvements of motor performance were observed in the age group 11–13 years old (19). In line with this finding, coordinative abilities can be trained particularly well in the period before puberty (20). At this age period, which is considered as the “golden age for motor development”, training should be oriented towards the technical aspects (21). The number of hours spent in individual practice as 11 years of age was a predictor of the competitive level they reached in their careers (22). When it concerns improvement of the passing skill, a 22-week training intervention focused on improving motor abilities and specific skills in 12 years old male soccer players found that execution time in a passing test decreased significantly in the intervention group (23); the differentiated training intervention they used were a combination of technical drills (e.g., passing, receiving and dribbling), small-sided games (SSG), game situations, and free play. Because of the large variation of the training program, it is difficult to point out which exercise(s) that gave the largest contribution to positive effect on the passing-skill acquisition.

In a recently published review, it was concluded that training using SSG has a positive effect on soccer players' short-passing abilities (24). Previously, training using 14 sessions focused on modified SSG significantly improved execution of passes during match play in youth male soccer players aged 10–12 years (25, 26). A key factor to improve the passing skill in youth soccer is the number of ball contacts and passes, and by using SSG with 3 or 4 players in each team a significantly increased number ball contacts and passes was registered compared to SSG teams with 6 or 8 players (27, 28). It appears that SSG, with few players, is a training context that potentially could improve passing skills in youth female soccer players. In contrast to training using SSG, where the players are being challenged to acquire movement solutions in side-foot kick passing technique appropriate for match conditions, soccer training using decontextualized exercises aims to improve important aspects of performance using structured and repetitive tasks starting with low-complexity exercises and thereafter transit to more complex and challenging contexts. Previously, we designed a passing-skill training (PST) concept consisting of five side-foot kick exercises and a theory lecture focusing on key factors for a side-foot kick with high accuracy; the elite female soccer players that were assigned to the intervention group improved their side-foot kick accuracy, whereas the control group did not show any improvements (29).

Despite the known benefits of SSG in skill development, limited research has been done on their comparative effectiveness against structured passing-skill training (PST) in youth female soccer players. This study aims to fill this void by evaluating the specific impact of these training modalities on passing accuracy. Therefore, the purpose of the study was to compare the effectiveness of a PST concept and three-a-side SSG on improving side-foot kick accuracy among youth female soccer players.

## Materials and methods

### Participants

Twenty youth female soccer players volunteered to participate in the study and the participants' legal guardian(s) gave their written informed consent to the players' participation in the study. Seventeen of the participants [age: 12.7 ± 0.8 years (mean ± standard deviation); body mass: 52.2 ± 7.9 kg; stature: 162 ± 5 cm; experience in soccer: 6.6 ± 1.6 years] completed the study. All participants belonged to the same team, which was ranked among the top 10 in Sweden within their age group. The team maintained a year-round training schedule, practicing three times per week. The test procedures were performed in accordance with the World Medical Association's Declaration of Helsinki—Ethical Principles for Medical Research Involving Human Subjects 2008, and the study was approved by the Regional Ethical Review Board, Uppsala, Sweden.

### Study design

To investigate the effects of the two training regimes, a pre-post-intervention study was implemented. Before the start of the training intervention, three side-foot kick tests were performed (pre-tests). Based on the results of the pre-tests, the participants were matched into two groups. The results in each test gave the participant a ranking from 1 to 20. The ranking points from the three tests were added and the sum was used for the subsequent matching procedure. The top ranked participant was assigned to group A. The participants ranked second and third were assigned to group B. The participants with ranking number 4, 6, 7, 9, 12, 14, 15, 17, and 20 were assigned to group A and consequently, participants ranked 5, 8, 10, 11, 13, 16, 18, and 19 were assigned to group B. Hence, both groups ranking sum is 105. Thereafter, the groups were randomly assigned to follow either the PST concept or training using three-a-side SSG in addition to their regular training. During the 4.5-week conditioning period, both groups performed nine 30-min training sessions as a part of the 75-min regular training that was organized twice a week. The remainder of the training had the focus to develop the functional technique, where decision-making was an important role in both attacking and defensive play. However, the regular training sessions during the intervention did not contain training using SSG. After the training intervention, the participants performed the three side-foot kick tests (post-tests) once more. A total of 17 participants (9 in the PST group and 8 in the SSG group) fulfilled the training requirements of at least 8 out of 9 training sessions and carried out the post-tests. The test results are based on the participants who completed the study.

### Testing procedures

The pre- and post-tests included three 10-m side-foot kick passing-accuracy tests with increasing level of complexity: (1) stationary ball with a fixed target (SBFT), (2) rolling ball with a fixed target (RBFT), and (3) rolling ball with a moving target (RBMT).The tests were performed at an indoor soccer field with artificial grass turf (XM 40, Fieldturf Tarkett, Nanterre, France). The same set of 15 pressured controlled balls (0.9 atm) of size 5 (Beau Jeu, Adidas AG, Herzogenaurach, Germany) was used. Each side-foot kick was preceded by a 3 s countdown (Stopwatch, Fitlb, San Jose, CA, USA) with one beep every second. The first beep was regarded as the start of the test and the last beep, with a higher tone, was regarded as the intended time of the kick. The computer program was set to have 10 s between kicks in each series. All three tests consisted of 20 side-foot kicks (4 series with 5 kicks/series) and the rest period between series was 40 s. All tests were recorded using video cameras (HC-V750, Panasonic, Osaka, Japan), that were positioned 3.5 m above the turf, to allow subsequent analyses of the side-foot kick passes' accuracy and speed (Dartfish TeamPro version 8, Dartfish HQ, Fribourg, Switzerland).

#### SBFT test

**Figure 1:**
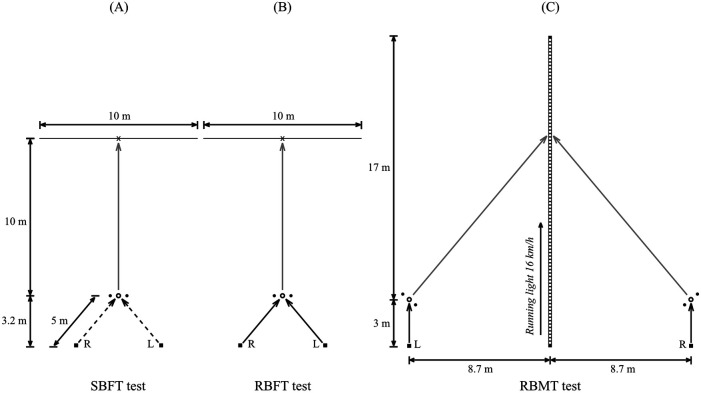
The arrangement of the three side-foot kick tests: **(A)** stationary ball with a fixed target (SBFT), **(B)** rolling ball with a fixed target (RBFT), and **(C)** rolling ball with a moving target (RBMT). Black dashed arrow represents the participant running without the ball; grey arrow represents the intended trajectory of the ball; black arrow represents the participant running with the ball; the cross sign represents the fixed target (i.e., the target stick with the diameter 35 mm and the length 1.7 m); R, start position when the side-foot kick was executed with the right foot; L, start position when the side-foot kick was executed with the left foot; and black rectangle with white dots represents the LED-strip.

In the first test, the participant's ability to accurately pass a stationary ball towards a fixed target (i.e., a 1.7 m high target stick with a diameter of 35 mm) was tested (Figure 1A). The run-up started 5 m from the pre-determined ball-strike position, with a fixed run-up angle of 40°. The participant was instructed to perform the side-foot kick within 3 s from the beep that started the countdown. The aim of each side-foot kick was to hit the target stick positioned 10 m from the position of the ball strike. To be considered as an acceptable kick, the ball had to roll on the artificial grass turf the whole 10-m distance. The first series was started with the right foot as striking foot; thereafter, the participant alternated between feet in each series. Hence, in the second series started with the participant performing the side-foot kick with the left foot. The accuracy was defined as the distance between the centre of target stick and the centre of the ball when it passed the target line (i.e., the line perpendicular to the fictive line between the ball's position at the kick and the target stick). Ball speed was calculated based on the ball's registered time between a marked line 2 m in front of the target line and the target line.

#### RBFT test

In the second test, the participant's ability to accurately pass a rolling ball (i.e., a ball on the move) towards a fixed target was tested (Figure 1B). The run-up with the ball started 5 m from the pre-determined ball strike position. The participant was instructed to move the ball in a straight path, from the start position to the ball-strike position, and execute the side-foot kick within 3 s from the beep that started the countdown. The rest of the execution and analyses of the RBFT test followed the same procedure as in the SBFT test.

#### RBMT test

In the third test, the participant's ability to accurately pass a rolling ball (i.e., a ball on the move) towards a moving target was tested (Figure 1C). The start position for the run-up was aligned with the start of the strip of light emitting diodes (LED) and positioned 8.7 m perpendicular to the straight 20-m LED-strip. The run-up with the ball started 3 m from the pre-determined ball-strike position during which the participant was instructed to move the ball in a straight path. The shorter run-up distance, compared to the RBFT test, was set to allow the participant to identify the moving target of a 0.2-m section (i.e., emulating the length of a teammates foot) of the lit LED-strip. The “running-light section” was started on the second countdown beep and its constant speed was set to 4.44 m/s (i.e., 16.0 km/h) simulating a running teammate. The starting time of the moving target were chosen with the intention that the passes are executed with approximately the same angle between run-up direction and ball trajectory as well as a passing length of approximately 10 m as in the preceding two tests. The participant was instructed to execute the side-foot kick within 3 s from the beep that started the countdown with the aim to hit the centre of lit-up section when the ball passed the LED-strip. In contrast to the other two tests, the first series of five side-foot kicks was performed using the left foot and thereafter the participant alternate striking foot between series. The accuracy was defined as the distance between the centre of the 0.2-m lit-up section and the centre of the ball when it crossed the LED-strip, and this position was also used to calculate the passing length (i.e., the distance from position of the ball strike to the position where the ball crossed the LED-strip). Mean ball speed was calculated as the quotient between passing length and the ball's registered time between ball strike and the LED-strip passage.

### PST concept

Before the start of the first training session included in the intervention, the participants in the PST group underwent a 30-min theory lecture focusing on key factors (e.g., position and direction of the support foot, the path of the kicking foot before the ball strike, foot angle at the ball strike, and point of the ball that should be hit) for a side-foot kick with high accuracy. During the lecture, they were also educated about potential faults that could influence the accuracy of the pass negatively. A repetition of the basics of the theory lecture was held before the fifth training session. Based on the content in the theory lectures, the participants were encouraged to reflect on misdirected passes under the training and what caused the poor accuracy. All participants in the PST group were given a printed version of the pictures included in the theory lecture as support in the reflection process. The participants brought the compendium to all training sessions.

**Figure 2:**
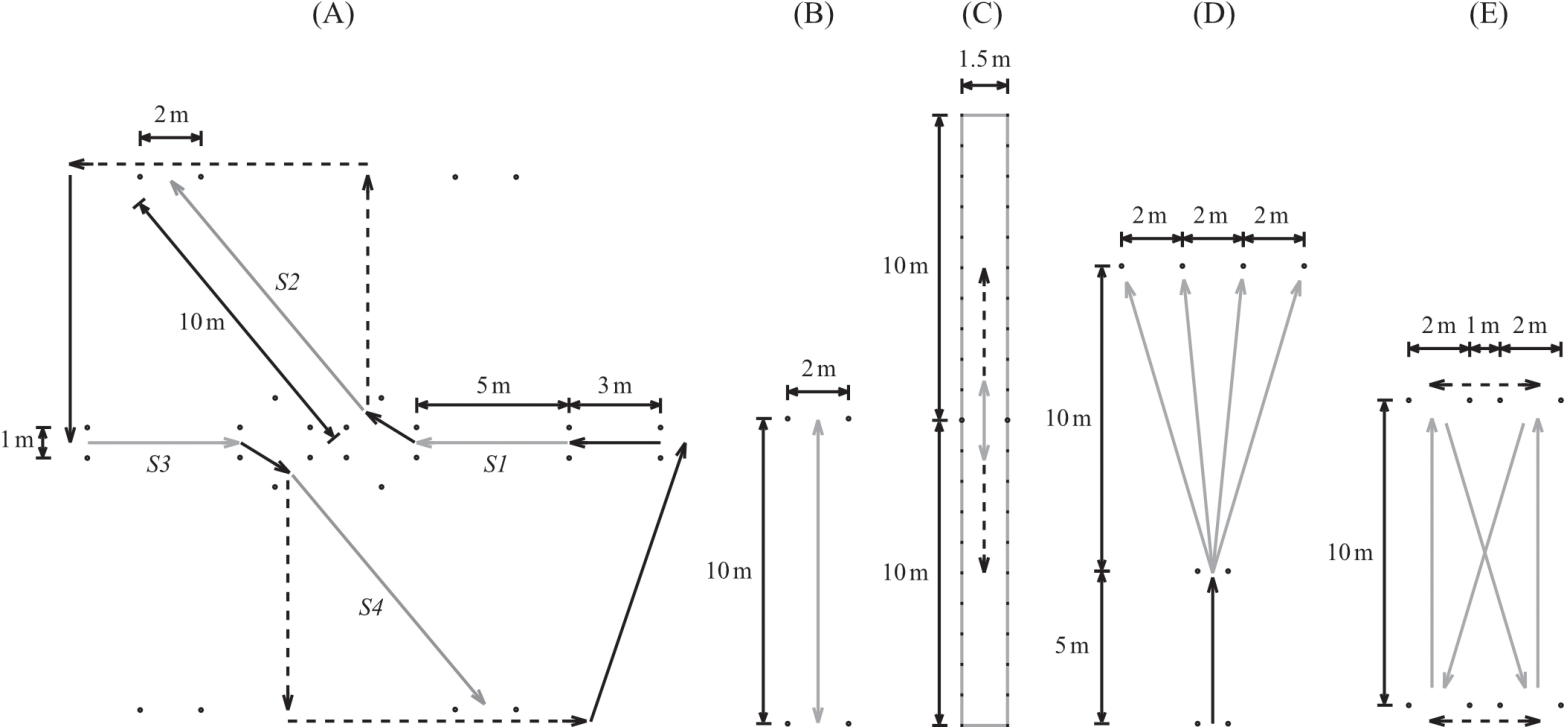
Graphical description of the five side-foot kick exercises **(A–E)** that were included in the passing-skill training (PST) concept. Black dashed arrow represents the participant running without the ball; grey arrow represents the trajectory of the ball; black arrow represents the participant running with the ball; and dots represent disc cones.

The intervention consisted of nine 30-min training sessions, that was scheduled before the start of the regular training. For each training, the participants executed five training exercises (5 min/exercise and 1 min rest between exercises) (Figures 2A–E), which were supervised by the same two coaches that gave internal-focus feedback on the execution of the side-foot kicks based on the content in the compendium. In all exercises, the participants were instructed to alternate between striking feet.

In the first exercise, all participants in the PST group were included simultaneously. The exercise consisted of four sections, where the ball is passed on the move by the participant to a teammate that receives the ball (Figure 2A). In section S1 and S3, the participant used a side-foot kick to pass the ball between two disc cones. In section S2 and S4, the participant that received the ball in the preceding section takes the ball with her and passes the ball between two disc cones to a teammate that is running on a straight line. At half time, the direction of the exercise was changed to alternate striking foot of the passes.

In the second exercise, two participants kicked the ball 10 m back and forth to one another, and they aimed to pass the ball between two disc cones that were 2 m apart (Figure 2B). Initially, the participants stopped the ball so that the side-foot kick was performed with a stationary ball. As the participants progressed, they used two-touch passing from training session five.

In the third exercise, two participants passed the ball between each other with one touch, trying to keep the ball within a 1.5-m wide corridor, where the lateral boundary lines were represented by ropes (Figure 2C). The participants started 2 m apart, and after each ten completed passes both participants backed 1 m (e.g., after 30 completed passes the participants were 8 m apart). If the ball crossed one of the ropes, the participants had to go back to the start position and make another try. Before training session five, the width of the corridor was reduced to 1.2 m.

In the fourth exercise, four 4-cm high disc cones were placed 2 m apart on a line 10 m from the position of the kick (Figure 2D). A ball was placed on each cone and the participant was instructed to hit the balls off the cones with as few side-foot kicks as possible. During the first four training sessions, the side-foot kicks were executed with a stationary ball. During the subsequent five training session, the distance from the start position to the ball-strike position was 5 m and the participant executed the side-foot kicks with the ball on the move.

In the fifth exercise, two pairs of disc cones with a 2-m gap between the cones in each pair were placed on two parallel lines 10-m apart (Figure 2E). There was a spacing of 1 m between the innermost cones in each pair. The two participants were positioned behind the cones on each side. One of the participants aimed to pass the ball straight between the two outer cones on each side. The receiver used two-touch side-foot kicks to pass the ball diagonally between the other participant's two outer cones on the opposite side. After the delivery of a pass, the participant moved sideways to receive the next pass. When one of the participants failed to pass the ball between the intended pair of cones, the participants switched tasks and the participant who kicked diagonally went to straight passing and vice versa. To increase the level of complexity, the players were instructed to use one-touch passing from training session five.

### Three-a-side SSG

The SSG group completed nine 30-min training sessions, that was scheduled before the start of the regular training, consisting of five 5-min blocks of three-a-side SSG with 1 min rest between blocks. The pitch size was 16 m × 8 m and the width of the goals was 0.8 m. The participants were instructed by the coach to focus on accurate passing and controlled receiving of the ball during the SSG. The coach also encouraged the participants to “use movement”, “support play”, and “create space”.

### Statistical analyses

Test results are presented as the means and standard deviations. The agreement of test variables with a normal distribution was assessed with the Shapiro–Wilk test. Paired and independent *t*-tests were employed to analyse within-group and between-group differences, respectively, providing a robust framework for detecting significant changes in passing accuracy. The effects size (*η*^2^) was calculated for each analysis and was interpreted as: small effect for 0.01 ≤ *η*^2^ < 0.06, moderate effect for 0.06 ≤ *η*^2^ < 0.14, and large effect for *η*^2^ ≥ 0.14 (30). All statistical analyses were assumed to be significant at an alpha level of 0.05. The statistical analyses were conducted using IBM SPSS Statistics software, Version 29 (IBM Corporation, Armonk, USA).

## Results

### Within-group differences

Results for the three side-foot kick tests before and after the training intervention are presented in Table 1. The paired-samples *t*-tests revealed that the PST group improved the accuracy for all three side-foot kick tests, SBFT (*t* = 4.59, *P* = 0.0018, *η*^2^ = 0.72), RBFT (*t* = 2.38, *P* = 0.044, *η*^2^ = 0.41), and RBMT (*t* = 3.55, *P* = 0.0075, *η*^2^ = 0.61), during the training intervention. No significant improvements in side-foot kick accuracy were found in the SSG group in the RBFT (*t* = 0.45, *P* = 0.67, *η*^2^ = 0.028) and RBMT (*t* = −1.22, *P* = 0.26, *η*^2^ = 0.18) tests; however, a significantly reduced accuracy was found for the SBFT test (*t* = −4.29, *P* = 0.0036, *η*^2^ = 0.72) in the SSG group.

**Table 1:** Results for side-foot kick tests before and after the 4.5-week training intervention.

Test	Variable	PST group		SSG group	
		Pre	Post	Pre	Post
SBFT	Δ	0.56 ± 0.14	0.43 ± 0.10**	0.52 ± 0.12	0.71 ± 0.17**
	*v*	8.4 ± 2.0	7.8 ± 1.2	7.9 ± 1.9	8.2 ± 1.5
RBFT	Δ	0.68 ± 0.24	0.48 ± 0.14*	0.66 ± 0.16	0.65 ± 0.14
	*v*	8.0 ± 1.7	7.9 ± 1.5	7.7 ± 1.5	7.9 ± 1.4
RBMT	Δ	0.97 ± 0.34	0.73 ± 0.27**	1.05 ± 0.34	1.20 ± 0.40
	*v*	9.6 ± 1.8	10.3 ± 2.0*	9.1 ± 1.1	9.9 ± 1.0*
	*l*	11.4 ± 0.8	11.2 ± 0.9	10.9 ± 1.2	11.7 ± 0.8

Values are expressed as mean ± standard deviation for the 10-m side-foot kick tests before (pre) and after (post) the 4.5-week training intervention. PST group, passing skill training group (*n* = 9); SSG group, small-sided game group (*n* = 8); SBFT, stationary ball with fixed target; RBFT, rolling ball with fixed target; RBMT, rolling ball with moving target; Δ, absolute deviation from the centre of the target (m); v, ball speed (m/s); l, passing length i.e., the distance from position of the ball strike to the position where the ball crossed the LED-strip (m). Paired-samples Student's *t*-test were used to investigate within-group differences between pre- and post-tests and the differences are reported as *for *P* < 0.05, **for *P* < 0.01.

There was an increased ball speed in the RBMT for both groups (PST: *t* = 2.57, *P* = 0.033, *η*^2^ = 0.49; SSG: *t* = 3.21, *P* = 0.015, *η*^2^ = 0.56) during the training intervention. However, there was no ball-speed differences for either group in the SBFT (PST: *t* = −0.96, *P* = 0.37, *η*^2^ = 0.12; SSG: *t* = 0.92, *P* = 0.39, *η*^2^ = 0.096) or RBFT (PST: *t* = −0.47, *P* = 0.65, *η*^2^ = 0.031; SSG: *t* = 0.64, *P* = 0.55, *η*^2^ = 0.049) test.

### Between-group differences

**Figure 3:**
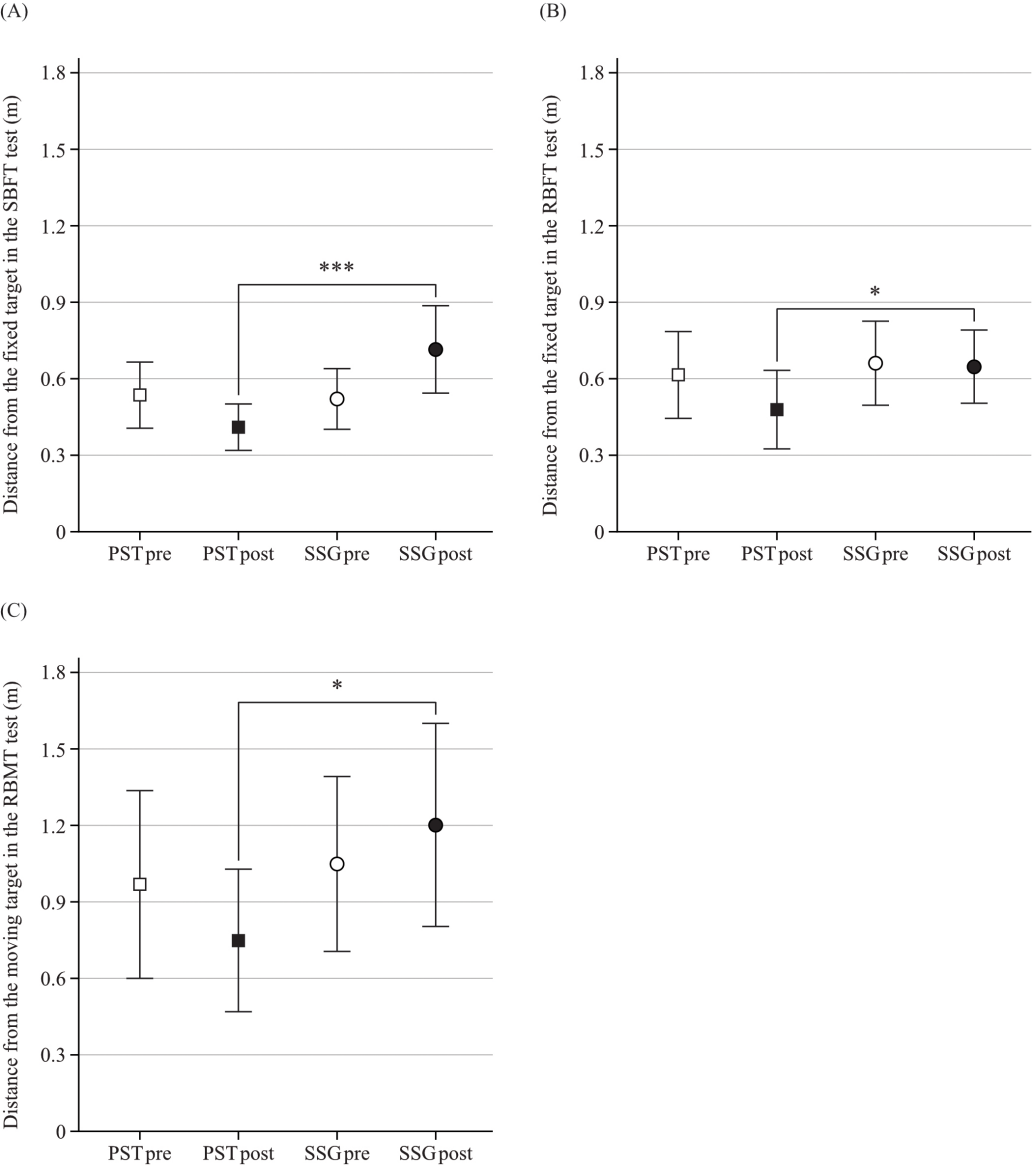
The accuracy of the three side-foot kick tests: **(A)** stationary ball with a fixed target (SBFT), **(B)** rolling ball with a fixed target (RBFT), and **(C)** rolling ball with a moving target (RBMT). The side-foot kick accuracy of each test is represented by the absolute distance between the target's centre and the ball's centre before (pre) and after (post) the 4.5-week training intervention, where filled/open squares and circles represent mean values of the passing-skill training (PST) group and small-sided games (SSG) group, respectively. Error bars represent ± 1 standard deviation. Significant between-group differences between training regimes are reported as: *P < 0.05, and ***P < 0.001.

At the pre-tests, no between-group difference was found for side-foot kick accuracy for either SBFT (*t* = −0.60, *P* = 0.56, *η*^2^ = 0.023), RBFT (*t* = −0.14, *P* = 0.89, *η*^2^ = 0.0013), or RBMT (*t* = 0.65, *P* = 0.46, *η*^2^ = 0.027) test. However, there were significant between-group differences for all three tests after the intervention, SBFT (*t* = 4.33, *P* < 0.001, *η*^2^ = 0.56), RBFT (*t* = 2.46, *P* = 0.027, *η*^2^ = 0.29), and RBMT (*t* = 2.82, *P* = 0.015, *η*^2^ = 0.35), where the side-foot kick accuracy was consistently better for the PST group (Figure 3).

No between-group difference was found for ball speed for either SBFT (pre: *t* = −0.51, *P* = 0.62, *η*^2^ = 0.017; post: *t* = 0.57, *P* = 0.58, *η*^2^ = 0.021), RBFT (pre: *t* = −0.46, *P* = 0.65, *η*^2^ = 0.014; post: *t* = 0.057, *P* = 0.96, *η*^2^ = 0.00022), or RBMT (pre: *t* = −0.69, *P* = 0.50, *η*^2^ = 0.031; post: *t* = −0.55, *P* = 0.59, *η*^2^ = 0.020) test. In the RBMT test, there were no between-group differences in passing length, either at pre-test (*t* = −1.08, *P* = 0.30, *η*^2^ = 0.072) or post-test (*t* = 1.18, *P* = 0.25, *η*^2^ = 0.085).

## Discussion

The purpose of the study was to compare the effectiveness of a PST concept and three-a-side SSG on improving side-foot kick accuracy among youth female soccer players. The results of the current study demonstrate that the participants in the PST group improved their side-foot kick accuracy significantly across all three passing tests, but no significant improvements in passing accuracy were found in the SSG group during the nine 30-min training sessions included in the 4.5-week training intervention.

Previously, we showed that short technique-intense training period focused on side-foot kick performance improved elite female soccer players' passing accuracy in the tests where the ball strike was performed on a moving ball (29); however, they did not improve the fundamental skill of passing a stationary ball towards a fixed target. In the current study, the participants in the PST group improved their side-foot kick performance in both tests where the task was to pass a stationary or moving ball towards a fixed target (Table 1). Worth noting is that after the intervention, the accuracy of the PST group in the SBFT test (0.43 ± 0.10 m) was in accordance with the accuracy reported for the elite female players (0.84 ± 0.19 m), because the distance from the ball-strike position to the target was twice as long for elite female players (20 m vs. 10 m). This observation was somewhat unexpected given that the elite female players were older and part of a Swedish first league team; however, the potential for improvement of the isolated skill of passing a stationary ball towards a fixed target is greater for youth female players as a result of their enhanced trainability of motor performance, coordinative abilities, and technical skills (19, 20).

The significant improvement of the accuracy of the PST group in both tests with higher complexity (i.e., where the participants passed a moving ball) is probably to some extent related to, the previously mentioned, enhanced trainability of the fundamental motor skills in youth soccer players. Development of basic fundamental motor skills serves as building blocks allowing for acquisition of more complex and difficult soccer-specific motor skills (31); therefore, it is suggested that youth soccer coaches should include training of fundamental motor skills before puberty with an emphasis on the quality of movement (31, 32). To develop motor skills, augmented feedback could be an important factor to improve an athlete's performance, and it should therefore be applied in sports training (33). In general, augmented feedback could be provided as external-focus feedback which is movement-effect related (also referred to as “knowledge of results”) or as internal-focus feedback which instead is body-movement related (also referred to as “knowledge of performance”), and previously it was showed that experienced soccer players' execution of lofted passes showed a greater accuracy after the external-focus feedback condition compared to the condition where the internal-focus feedback was used (34). In line with this finding, it was reported that external-focus feedback was more effective in learning of soccer “head kick” among youth female soccer players (35). In the current study we used a combination of internal-focus and external-focus feedback; the coaches provided internal-focus feedback based on the participants' side-foot kick performance during the passing-skill training, whereas the participants in the PST group received direct external-focus feedback on their passing accuracy using the task constraints (i.e., cones and ropes) as reference. A recent review supports the use of a combination of prescriptive external-focus feedback (including information about what to do next to improve) and internal-focus feedback if the aim is to enhance motor-skill learning (36). Hence, the feedback given to the PST group could be one contributing factor to the significant improvement of the side-foot kick accuracy.

Training using SSG is a frequently used method with the aim to improve physiological abilities and technical skills in youth soccer (37). In the current study we used SSG with 3 vs. 3, and previously three-a-side SSG resulted in higher mean heart rate, greater involvement with play, more dribbling, and more short and medium passes than five-a-side SSG in youth male soccer players (38). An 8-week training intervention, consisting of SSG with 3 vs. 3, improved youth male soccer players' maximal aerobic power and overall soccer-specific technical skills (i.e., sum of juggling, dribbling, heading, passing, and shooting skills) (39). It has been reported that SSG simulate the overall movement pattern in elite women's soccer matches and the players are, therefore, challenged to practise their short-passing skills during SSG (40). Therefore, it can be expected that the SSG group would improve their general short-passing abilities. However, in contrast to the PST group, the SSG group did not improve the passing accuracy for any of the three tests (Table 1). In a recent review, it was stated that SSG has a positive impact on soccer players' short-passing abilities (24), but this statement was based on the results from only two studies which used the Loughborough Soccer Passing Test (LSPT) to analyse the short-passing ability (41, 42). Both these studies used the overall skill performance time (i.e., execution time + penalty time) to verify the SSG's positive impact on short-passing abilities. The “execution time” is the time to complete 16 passes against target areas attached on four benches that are placed different directions and perpendicular to the player that is tested. For the variable “penalty time”, time is added for missing the bench, missing the target area, handling the ball, passing the ball from outside the designated area, ball touching a cone, and performing the test over 43 s. Only the two first two penalty components, together with a bonus time awarded if the player hit the middle 10-cm strip of the target area, reflect the side-foot kick accuracy. Consequently, none of the three variables that normally are reported for LSPT performance is fully appropriate to detect side-foot kick accuracy of soccer players. A previous study highlighted this limitation, where it was shown that execution time of the LSPT did not differentiate selected and de-selected male youth soccer players (10–18 years), but the talented players were generally more accurate in their passing and ball control (i.e., a reduced penalty time) compared to their less-skilled counterparts (43); the authors concluded that having superior passing accuracy is eventually a good predictor for future performance level.

Based on the enhanced trainability of motor skills in youth soccer players, it is concerning that the fundamental ability to execute accurate side-foot kicks on a stationary ball towards a fixed target in the SBFT test declined significantly in the SSG group and possibly due to the training using SSG. Short passes during SSG are generally executed on a moving ball, but this should not be a reasonable explanation to the demonstrated decline in passing accuracy. One potential explanation to this result is that the main focus in SSG is not the accuracy; instead, the aim is to keep the ball within the team and the teammates compensate for an inaccurate pass by adjusting their movement direction and/or speed. Moreover, the general passing length during three-a-side SSG using a small pitch is relatively short (27), therefore, a somewhat inaccurate pass will not negatively affect the teammate's chance to receive the ball. The visual feedback the player may receive from the situation is that the teammate successfully received the pass, and the pass's accuracy was sufficient. Conversely, the participants in the PST group receive instant external-focus feedback on the accuracy of every single side-foot kick through the task constraints (cones or boundary lines) that are a part of the setup in all five exercises (Figures 2A–E). As previously argued in the study with elite female soccer players, the external-focus feedback based on the outcome of each side-foot kick could be one of the key factors behind the improvements of the side-foot kick accuracy (29). Also, the self-reflection of the passing quality, based on the information in theory lectures and the compendium, may have contributed to the significant improvements in the PST group. Hence, passing-quality reflections are rarely a conscious part of daily training, because the teammates adapt to the ball's trajectory and speed, filtering out inaccurate passes.

The previously developed and to the current study refined training concept had a positive effect on the PST group's side-foot kick accuracy in the RBMT test, which is the most complex and match-like test where the LED-strip's “running light” simulates a running teammate. The importance of a player's ability to accurately pass the ball to a running teammate was previously shown in a study analysing passing actions; they found that the efficiency of the pass, the trajectory of the receiver, and where on the pitch the pass was received were the main performance indicators for success in completing the play by a shot (44). Training using SSG with few players in each team induces many ball contacts and short passes (27, 28), which should entail that the ability to perform accurate side-foot kick passes on a rolling ball to a teammate who is moving is challenged. However, the SSG group did not improve the side-foot kick accuracy in the RBMT test. On the contrary, the improvement of the accuracy in the PST group was somewhat unexpected since their main training focus were not to pass teammates on the move, and an interesting finding in this context is that the improved accuracy was accompanied with an increased ball speed (Table 1). This finding is in contrast to the previously reported speed/accuracy trade-off in soccer, which leads to a reduced ball speed when an accuracy demand is introduced (45, 46). Hence, the PST concept appears to have a positive influence on the ability to accurately pass the ball on the move to a moving target with a significantly increased ball speed, which definitely is an important factor for success in soccer as reflected by a continuous increase of passing ball speed in the World Cup during the period 1966–2010 (47).

Consistent with prior studies (23, 29), our findings suggest that targeted technical training like PST can offer more controlled and focused skill enhancement than SSG, aligning with literature emphasizing the importance of deliberate practice in skill acquisition (22). Hence, the results of the current study advocate for soccer coaches to integrate more PST sessions into their regular training schedules, especially when the aim is to enhance passing accuracy among youth female players.

The current study investigates the impact of a 4.5-week long training period on passing accuracy among youth soccer players and the relatively short duration of the intervention is a limitation. Therefore, it would be valuable to investigate whether a prolonged training period leads to further improvements in passing accuracy. To make the test with the LED-strip even more authentic to a match situation, where the player should time the direction and speed of the pass to an imagined running teammate, a possibility to vary the speed of the “running light” to simulate teammates with different movement speeds would be preferrable. The abilities to adapt the face angle of the kicking foot and the applied force to the ball are key factors for soccer players to master and these factors place high demands on the coordinative skills. Therefore, passing-skill training should be a natural part of the regular training among youth soccer players, that have great opportunities develop the side-foot kick accuracy as a consequence of the high trainability of their coordinative abilities (20). Because of the significant improvements the PST group displayed, it would have been of interest to try to quantify the impact of each of the key elements in the PST concept (i.e., theory lectures, passing-skill training, external-focus and internal-focus feedback during training). Further studies are also warranted to investigate the influence of sex, retention of the training effect, and dose-response relationship between training volume and improvement of side-foot kick accuracy. In this context, it would naturally have been of great interest to investigate the impact of the PST concept on match performance to analyse if, for example, the passing success rate improves with passing-skill training or not.

## Conclusions

Results in the current study indicate that the PST concept significantly enhances passing accuracy across all tested variables, unlike SSG, suggesting PST's superiority in technical skill development. It appears that passing exercises with task constraints (i.e., cones and ropes) in addition to theory lectures focusing on key factors for a side-foot kick with high accuracy, gives players the foundation to do self-reflections on their passing accuracy and consider plausible causes to misdirected passes. Therefore, soccer coaches should integrate the PST concept into their regular training schedules, especially when the aim is to enhance passing accuracy among female players.

## Data Availability

The raw data supporting the conclusions of this article will be made available by the authors, without undue reservation.
